# Development of Sarcopenia Management Systems for Former Homeless Older Adults Residing in Supportive Housing: Mixed Methods Study

**DOI:** 10.2196/95347

**Published:** 2026-07-29

**Authors:** Phatcharaphon Whaikid, Noppawan Piaseu, Tiraporn Junda, Kamonrat Kittipimpanon, Anita M Souza, Kulapong Jayanama

**Affiliations:** 1 Doctor of Philosophy Program in Nursing Science (International Program), Faculty of Medicine Ramathibodi Hospital, Faculty of Nursing, Mahidol University, Bangkok, Thailand; 2 Department of Community Health Nursing, Ramathibodi School of Nursing, Faculty of Medicine Ramathibodi Hospital, Mahidol University, 270 Rama 6 Road, Bangkok, 10400, Thailand, +6622012895; 3 Department of Biobehavioral Nursing and Health Informatics, School of Nursing, University of Washington, Seattle, WA, United States; 4 Faculty of Medicine Ramathibodi Hospital, Mahidol University, Bangkok, Thailand

**Keywords:** sarcopenia, homelessness, older adults, supportive housing, community health systems, Thailand

## Abstract

**Background:**

Former homeless older adults represent a highly vulnerable population facing substantial community health challenges, including an elevated risk of sarcopenia due to compounded health and social disadvantages. Developing a context-specific management system is crucial for enhancing early detection, prevention, and care within supportive housing settings.

**Objective:**

This study aimed to develop and evaluate the feasibility of a sarcopenia management system for former homeless older adults in Thailand.

**Methods:**

mixed methods design was used, consisting of three phases: (1) development, (2) implementation, and (3) preliminary evaluation testing. The development phase engaged 18 stakeholders and 32 focus group participants (former homeless older adults and staff) through 2 rounds of consultation. The system was subsequently implemented and preliminarily evaluated for feasibility, perceived quality, perceived benefits, and user satisfaction.

**Results:**

Through qualitative methods and a systematic review, a context-specific sarcopenia management system was developed for former homeless older adults. The system comprised five core components screening, classification, management, follow-up, evaluation and was integrated into routine care processes involving multidisciplinary staff and homeless volunteers within a supportive housing setting. The system was subsequently implemented and preliminarily tested among 116 former homeless older adults living in a supportive housing facility in Thailand. Preliminary findings indicated good feasibility and acceptability of the system. The perceived quality of the system achieved a mean score of 2.6 (SD 0.48), while perceived benefits were rated at 3.8 (SD 0.40). Overall satisfaction with system use and implementation processes achieved mean scores of 2.8 (SD 0.41) and 2.72 (SD 0.79), respectively, indicating high user satisfaction.

**Conclusions:**

This study presents the first context-sensitive sarcopenia management system specifically developed for former homeless older adults in supportive housing in Thailand. The system was well accepted and shows promise for strengthening early detection, prevention, and management of sarcopenia in community-based housing settings.

## Introduction

Sarcopenia has become a pressing public health concern amid global population aging, characterized by a progressive and generalized loss of skeletal muscle mass and function [1]. This condition not only heightens the risk of physical disability [2,3] and frailty [4] but is also linked to adverse outcomes such as falls [5], fractures [6], hospitalization [7], and mortality [8]. Previous studies have identified age, low physical activity, malnutrition [9], and poor diet quality [10] as major risk factors for sarcopenia.

Among vulnerable populations, former homeless older adults residing in supportive housing represent a group in whom the prevalence of sarcopenia is likely underestimated and inadequately addressed. Prolonged exposure to poverty, multimorbidity [11], chronic diseases [12,13], persistent health disparities, and limited access to health care services place individuals with a history of homelessness at substantially elevated health risks [14,15]. As a result, this population experiences accelerated aging and premature morbidity, often being classified as older adults at approximately aged 50 years, which is markedly earlier than the general population, typically defined as 60‐65 years, depending on national criteria [16]. This premature aging trajectory contributes to profound health inequities, increased vulnerability to sarcopenia, and a heightened risk of early mortality from largely preventable or manageable conditions [12,17]. Notably, the average age at death among individuals with lived experience of homelessness is approximately 51.6 years, nearly 2 decades younger than that observed in other socioeconomically deprived populations [18].

Despite this elevated vulnerability, sarcopenia among former homeless older adults frequently remains underrecognized and inadequately managed, particularly within supportive housing settings where health and social care resources are constrained. In community-based environments, early identification of sarcopenia is often limited by limited access to body composition assessment tools, such as bioelectrical impedance analysis (BIA), which reduces the feasibility of conventional diagnostic approaches. To address these practical challenges, the Asian Working Group for Sarcopenia (AWGS) 2019 introduced the concept of possible sarcopenia to facilitate early screening and timely intervention in nonclinical and resource-limited settings [19].

Although community-oriented screening frameworks such as the AWGS criteria are available, screening alone is insufficient to address the complex and ongoing needs of formerly homeless older adults. What remains largely absent is a structured, integrated management system that translates early identification into coordinated prevention strategies, personalized interventions, and sustained follow-up within supportive housing contexts. The lack of such a system limits the ability of health and social care providers to deliver proactive, consistent, and context-appropriate care, thereby perpetuating avoidable functional decline and health inequities in this underserved population. Accordingly, there is a critical need to develop a context-sensitive sarcopenia management system tailored to the realities of supportive housing settings. Such a system has the potential to strengthen early detection, support timely and individualized interventions, and promote functional independence and well-being among former homeless older adults. Therefore, this study aimed to develop and evaluate a sarcopenia management system for former homeless older adults residing in a supportive housing facility in Thailand.

## Methods

### Study Design

This study used an embedded mixed-methods design [20] to develop and evaluate a sarcopenia management system for former homeless older adults residing in a supportive housing facility in Thailand. The design was selected to support system development within a real-world institutional context, where health care delivery is shaped by organizational structures, staff-led processes, and resident dependency. Qualitative and quantitative components were integrated across three sequential phases: system development, implementation, and preliminary evaluation. Qualitative methods were used to explore contextual needs, operational challenges, and stakeholder perspectives, informing the design and refinement of system components. Quantitative methods were subsequently applied to assess the feasibility, acceptability, and perceived performance of the developed system during pilot implementation [21]. The overall development and evaluation process was guided by Donabedian’s framework [22], which conceptualizes health care quality through interrelated dimensions of structure, process, and outcomes. This framework provided a systematic approach to designing a context-sensitive management system that aligns organizational resources and care processes with measurable outcomes relevant to sarcopenia prevention and management in supportive housing settings. Purposive sampling was used to select the supportive housing facility and to recruit participants across 2 groups. For the preliminary evaluation phase, former homeless older adults aged 50 years or older who had resided in the facility for at least 6 months were eligible for inclusion. Individuals with cognitive impairment, defined as a Chula Mental Test (CMT) score below 15, or those unable to provide written informed consent, were excluded. For the system development phase, stakeholders, including administrators, staff, first-aid personnel, and support staff aged 18 years or older with proficiency in Thai, were recruited purposively from 6 of the 7 dormitories within the facility. The study was conducted at a government-operated supportive housing facility in Nonthaburi Province, Thailand, which provides shelter, basic health care, and rehabilitation services for individuals experiencing homelessness.

### Study Procedure

This study was conducted between March 2024 and March 2025 and followed a 3-phase, embedded mixed-methods approach comprising system development, implementation, and preliminary evaluation. The study procedure was designed to integrate evidence from systematic reviews, previously published qualitative findings, and stakeholder engagement to develop and test a context-sensitive sarcopenia management system for former homeless older adults residing in supportive housing. Purposive sampling was used to recruit participants who met the predefined eligibility criteria for each study phase.

### Phase 1: System Development

Phase 1 focused on developing the sarcopenia management system using multiple sources of evidence, including 2 previously published systematic reviews and 1 qualitative study.

First, findings from 2 systematic reviews were synthesized to inform system priorities and intervention selection [23,24]. The first review identified key personal and health-related factors associated with sarcopenia among older adults in Thailand, including BMI, waist circumference, type 2 diabetes mellitus, mild cognitive impairment, and depression [23]. These findings informed risk identification and screening priorities within the system. The second review examined the effectiveness of protein supplementation combined with resistance exercise for sarcopenia rehabilitation in community settings and demonstrated improvements in muscle mass and strength [24]. These findings guided the selection of intervention components suitable for implementation in supportive housing.

Second, the development process was informed by previous qualitative findings [21]. This qualitative inquiry explored lived experiences, perceptions, and contextual challenges related to sarcopenia among former homeless older adults, as well as perspectives of staff involved in their care. Key themes highlighted barriers to sarcopenia prevention and management, including limited health knowledge, constrained physical activity opportunities, nutritional challenges, and structural limitations within institutional care settings. These qualitative insights provided critical contextual understanding that complemented the systematic review evidence and directly informed the design and contextual adaptation of the system components [21].

Third, stakeholder engagement was integrated into the development process. In this study, stakeholders were defined as administrators, general staff, first aid staff, chefs, and homeless volunteer representatives who were former homeless residents serving in caregiving and supervisory roles within each dormitory. Thirty stakeholders participated in 2 structured meetings to validate system components, identify feasible care processes, and refine implementation strategies. This phase established the system’s framework, building on insights from the prior qualitative study, particularly the 2 overarching themes, “Insufficiency” and “The Price of Plenty” [21]. Accordingly, the implementation plan emphasized practical and context-sensitive strategies to promote dietary protein intake, oral health, and physical activity engagement. Sustainable food-based protein strategies included Nile tilapia cultivation and soy milk production, alongside resistance exercise training as the core physical activity component.

### Phase 2: System Implementation

Phase 2 involved implementing the sarcopenia management system in supportive housing. Implementation activities included three major components: (1) Training for sarcopenia screening, (2) implementing sarcopenia screening and classification, and (3) implementing interventions.

1. Training for sarcopenia screening: This process involved engagement of 18 staff members and 12 homeless volunteers. There were 2 main training sessions, including assessment of the 5-item Mini Sarcopenia Risk Assessment (MSRA) and measurements of calf circumference, handgrip strength, and gait speed. The research team also developed educational materials, including resistance exercise demonstration videos, and provided training on sarcopenia screening and exercise techniques.
2. Implementing sarcopenia screening and classifying sarcopenia risk and possible sarcopenia according to the AWGS 2019 criteria. This part recruited 116 older adults aged 50 years and above who had resided in supportive housing for at least 6 months and met the inclusion criteria for independence in Activities of Daily Living and stable chronic physical or mental health conditions. Impaired cognitive function, as defined by a CMT score below 15, was excluded. Screening and classification of sarcopenia risk and possible sarcopenia were then conducted according to the AWGS 2019 criteria.
3. Implementing interventions, including resistance exercise, Nile tilapia cultivation, and soy milk as locally feasible strategies to improve access to affordable dietary protein.

Participants engaged in a supervised resistance exercise program for 8 months, 5 days per week, with each session lasting approximately 45 minutes. The exercise program was led by trained staff and supported by instructional videos to ensure safe and consistent implementation. Participants also received nutritional support through soy milk supplementation. A hands-on demonstration was conducted to guide participants through each step of soy milk production. Soy milk was prepared and provided once per month for 8 consecutive months and served after resistance exercise sessions as a locally available dietary protein source. In addition, 2 former homeless older adult volunteers were assigned as tilapia cultivation leaders and were responsible for daily fish care, basic maintenance, and coordination with staff for harvesting and distribution to older adults and other former homeless older adults. Nile tilapia cultivation was maintained continuously for 8 months, and the fish were harvested periodically and prepared as meals, serving as a sustainable, locally available source of dietary protein to support the sarcopenia management program.

### Phase 3: Preliminary Evaluation

The preliminary evaluation phase included 116 homeless older adults, similar to Phase 2. Based on system implementation processes, the preliminary evaluation included perceived benefits, perceived quality, and satisfaction of the sarcopenia management system as assessed by staff members and former homeless older adults. Findings from this phase were used to inform the refinement of system components and assess readiness for broader application.

**Figure 1.**
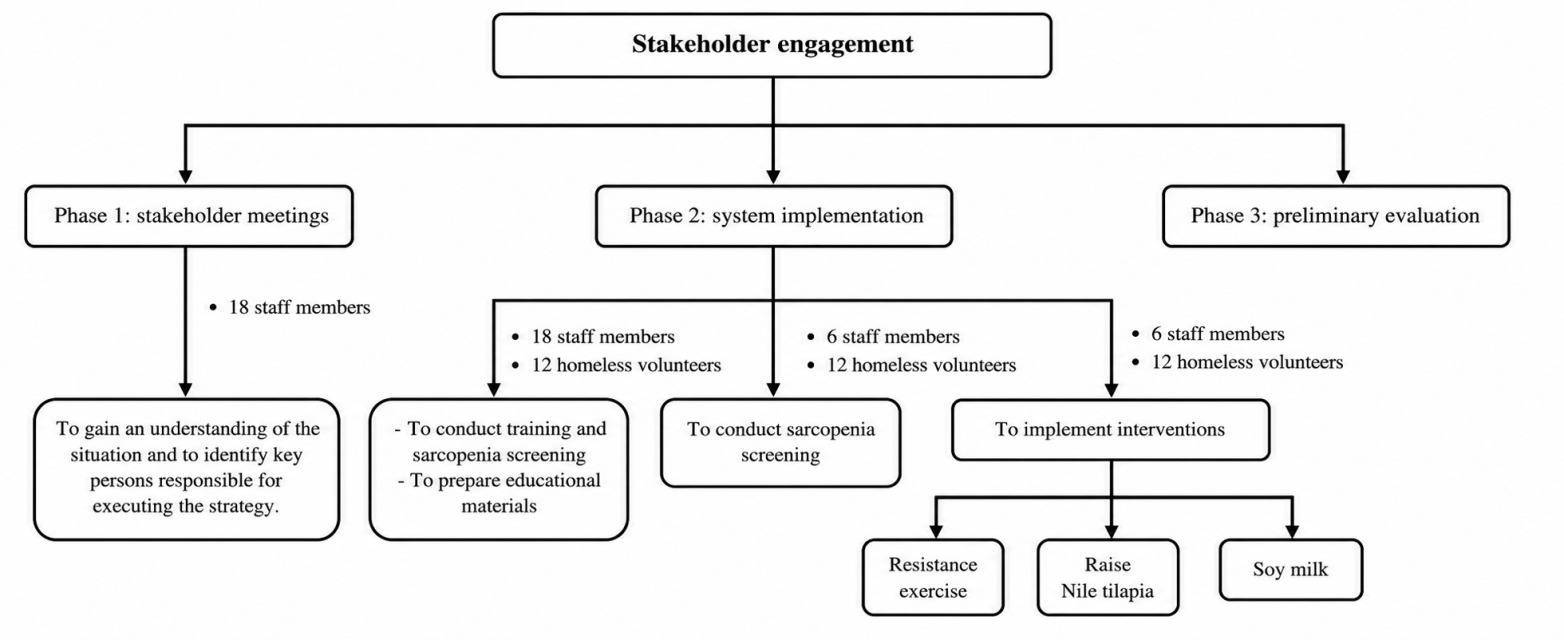
Development process of the sarcopenia management system.

The development process for the sarcopenia management system, through stakeholder engagement, for homeless older adults in supportive housing, is illustrated in Figure 1.

### Setting

This study was conducted at a supportive housing facility located in Nonthaburi Province, Thailand. The facility serves as a pivotal institution for vulnerable populations from both Nonthaburi and Bangkok. It was purposively selected because of its central role in addressing homelessness, particularly in Bangkok, which reports one of the highest rates of homelessness in the country. The supportive housing operates as a closed and structured system that provides temporary shelter, basic health care, and rehabilitation services for individuals experiencing homelessness, vagrancy, begging, or posthospital discharge. Its primary mission is to prepare former homeless individuals for reintegration into their families and communities while addressing their immediate physical, psychological, and social needs.

### Organizational Structure and Dormitory Arrangement

Administratively, the facility is organized under a hierarchical management structure comprising 1 head administrator, administrators, first aid staff members, 11 general staff members, and chefs, all of whom support daily operations and resident care. The facility is divided into 7 dormitories that accommodate residents based on functional status and daily activity patterns.

For the purposes of this study, 6 of the 7 dormitories were included in stakeholder engagement, focus group discussions, and implementation activities. Dormitory 7 was excluded because it primarily housed older adults who regularly worked outside the facility and spent substantial portions of the day away from the home. As a result, former homeless people in this dormitory had limited exposure to routine institutional care processes and intervention activities. To ensure contextual consistency and relevance to the sarcopenia management system, data collection and implementation activities were therefore conducted in the remaining 6 dormitories.

### Research Instruments

The research instruments used in this mixed-methods study included both qualitative and quantitative tools, applied across the 3 phases. In phase 1 (system development), qualitative data were collected through stakeholder consultations using semistructured guiding questions to identify priorities and refine system components. In phase 2 (system implementation), quantitative instruments were used for participant screening and sarcopenia assessment and classification, including the CMT, Barthel ADL Index, Modified MSRA, calf circumference, handgrip strength, gait speed, and BIA when feasible. Qualitative feedback from stakeholders was also obtained during implementation through structured consultations and brief semistructured interviews to support process evaluation and system refinement. In phase 3 (preliminary evaluation), researchers developed quantitative questionnaires to assess perceived benefits, quality, and satisfaction with the sarcopenia management system.

#### Screening Tools for Participant Recruitment

The following instruments were used as eligibility screening tools to assess cognitive function and functional status prior to enrollment in the study. Participants who did not meet the predefined criteria were excluded.

1. The CMT, developed by Jitaphankul [25], was used to assess cognitive function disorders among former homeless older adults. The test comprised 13 items with 19 possible answers, yielding scores ranging from 0 to 19. Each item was scored as 0 for an incorrect response and 1 for a correct response. A score of less than 15 on the CMT indicated the presence of a cognitive function disorder. In this study, participants were required to achieve a CMT score of 15 or higher to advance to the next phase of the research. For those who scored below 15, their information was shared with stakeholders for further assessment and follow-up.
2. The Barthel ADL Index, developed by Jitaphankul [26], was used to evaluate ability to perform daily activities. This index consisted of ten questions, with a total score ranging from 0 to 20. Each score reflected a specific level of dependence, providing valuable insights into the participants’ functional abilities. Scores between 0 and 4 indicated total dependence, while scores from 5 to 8 represented severe dependence. Participants who scored between 9 and 11 were classified as having moderate to severe dependence, whereas those scoring between 12 and 20 were considered minimally dependent.

#### Instruments for Early Detection and Classification of Sarcopenia

The identification and classification of sarcopenia in this study were based on the AWGS 2019 criteria [19]. In the supportive housing setting, screening and classification primarily focused on identifying sarcopenia risk and possible sarcopenia using calf circumference, handgrip strength, and gait speed. BIA was incorporated into the management algorithm as a confirmatory assessment for suspected sarcopenia or severe sarcopenia when feasible or when referral to health care services was indicated. According to the system classification, participants were categorized into three groups: green (no sarcopenia risk), yellow (case finding or possible sarcopenia), and red (sarcopenia or severe sarcopenia). The selected assessment tools are established methods recommended by the AWGS 2019 criteria and are widely used in older adult populations to assess sarcopenia risk, muscle strength, physical performance, and muscle mass [19].

1. Screening tool for identifying sarcopenia risk: the researchers used the Modified MSRA, a 5-item instrument initially developed by Rossi et al [27]. The 5 items have possible scores ranging from 0 to 34, and a total score < 30 indicates a risk of sarcopenia. The MSRA assessment includes the following five components: (1) age, (2) the number of hospitalizations in the last year, (3) the level of physical activity, (4) regular meal consumption, and (5) weight loss in the last year. The questionnaires were translated from English to Thai by Akarapornkrailert (2020). The content validity index (CVI) for the MSRA 5-item questionnaire was established at 1.0 [28].
2. The screening tool for identifying possible sarcopenia was based on the criteria of the AWGS 2019 [19]. These assessments involve measuring calf circumference, handgrip strength, and gait speed. Sarcopenia management is based on the surveillance, maintenance, and reversal classification. The insights from the qualitative part guide the development of a management program combined with a thorough review of relevant literature as follows: (1) the green group refers to older homeless adults who are not at risk of developing sarcopenia following the use of MSRA assessment, (2) the yellow group refers to older homeless adults identified as case-finding or possible sarcopenia following CC assessment, handgrip strength assessment, and gait speed assessment, and (3) the red group refers to older homeless adults diagnosed with sarcopenia and severe sarcopenia.
  1. Calf circumference was measured using a standard tape with 1 millimeter precision. Participants sat with their knees bent at 90 degrees while the tape was placed around the widest part of the calf, parallel to the floor, and pulled taut. The measurement was recorded in centimeters, with values <34 cm for men and <33 cm for women indicating a case finding.
  2. Muscle strength was measured using a digital handgrip dynamometer, calibrated to ISO standards. Participants sat comfortably and gripped the dynamometer with their dominant hand, keeping the elbow at 90 degrees and the arm away from the trunk. After exerting maximal force, the test was performed twice, and the highest value was recorded in kilograms (kg). Handgrip strength below 28 kg for men and 18 kg for women indicated low muscle strength.
  3. Physical performance was assessed by measuring walking speed over a 6-meter distance using a stopwatch and a tape measure. The researcher first demonstrated walking at a normal pace, then participants stood at the starting point. On the “start” signal, the timer began and stopped when the participant reached the end. The time taken to walk 6 meters was recorded in seconds. Walking speed was calculated as distance/time (eg, 6 meters / 9 s=0.67 m/s). A walking speed ≤1 m/s indicated low physical performance.

#### Instruments for Preliminary Evaluation of the Sarcopenia Management System

These instruments were used to assess the quality, perceived benefits, and satisfaction associated with the sarcopenia management system among former homeless older adults and stakeholders.

1. Demographic and health questionnaire for older homeless adults: the questionnaire was developed from literature reviews by researchers. There are questions, including age, gender, years of homelessness among former homeless older adults in supportive housing, education level, the presence and type of chronic diseases, and nutritional status, which were assessed using the Short Form Mini Nutritional Assessment (MNA-SF). Additional health-related information included weight loss, smoking status, history of falls, and physical activity levels.
2. Benefits of the sarcopenia management systems questionnaire: the questionnaire was developed by the researchers based on an extensive review of relevant literature and was designed to assess the perceived benefits of the sarcopenia management system. The instrument included items addressing the benefits of sarcopenia screening, follow-up, maintenance, and reversal, as well as the overall benefits of the service. Participants rated each item on a 4-point Likert scale: excellent, good, fair, and needs improvement. In addition, an open-ended question was included to elicit participants’ opinions and suggestions on the system’s benefits. The Content Validity Index (CVI) was 1.00.
3. Quality of sarcopenia management systems questionnaire: the questionnaire was developed by researchers based on an extensive review of relevant literature and was designed to assess the quality of the sarcopenia management system. The instrument included domains related to process quality, staff quality, and overall service quality. Participants rated each item on a 3-point Likert scale: beyond expected, as expected, and below expected. In addition, an open-ended question was included to allow participants to provide comments and suggestions on the system’s quality. The CVI was 1.00.
4. Satisfaction of the sarcopenia management systems questionnaire: the questionnaire was developed by researchers based on an extensive review of relevant literature and was designed to assess the satisfaction of staff members and former homeless older adults following their use of the sarcopenia management system. The instrument included domains related to satisfaction with staff providing services, satisfaction with facilities, and overall service satisfaction. Participants rated each item on a 3-point Likert scale: beyond expected, as expected, and below expected. In addition, 1 open-ended question was included to elicit participants’ opinions and suggestions regarding the system. The CVI was 1.00.

### Data Analysis

Data were organized and analyzed separately for each phase. In phase 1 (system development), findings from 2 previously published systematic reviews and 1 qualitative study were synthesized narratively and integrated with data from stakeholder consultations to inform the design and contextual adaptation of the sarcopenia management system. Qualitative data from stakeholder consultations were summarized according to key themes related to system priorities, feasibility, and implementation strategies. In phase 2 (system implementation), process-related qualitative feedback obtained from structured consultations and brief semistructured interviews was reviewed and summarized to support ongoing refinement of the system. Quantitative screening and assessment data were used descriptively to classify participants according to the AWGS 2019 criteria and guide individualized management. In phase 3 (preliminary evaluation), the researchers analyzed data using SPSS 21.0 (Mahidol University). First, the raw data were reviewed, and the data entry process was checked for missing values and inconsistencies by performing frequency analysis to ensure data quality. Next, the sarcopenia screening (including calf circumference, gait speed, handgrip strength, and appendicular skeletal muscle index [ASMI]) was analyzed using descriptive statistics, including frequency, percentage, mean (SD), and range. The efficiency of the sarcopenia service system was evaluated using two questionnaires: one assessing the benefits of the service and the other evaluating its quality, both using frequency and percentage analyses. Finally, satisfaction with the sarcopenia service system was analyzed in a similar manner, using frequency and percentage.

### Ethical Considerations

In this research study, human research ethics were considered and approved by the research committee of the Faculty of Medicine at Ramathibodi Hospital, Mahidol University (approval number MURA2024/64). The primary researcher introduced herself with the research authorization letter and requested permission to collect data from the Department of Social Development and Welfare. Data collection was conducted strictly in accordance with ethical principles, with the researcher explaining the study’s objectives and the steps involved to participants. Participants were informed of their right to withdraw from the study at any time, without needing to provide a reason. Additionally, all data were kept confidential unless the participant authorized its disclosure. To ensure privacy, participants were assigned fictitious names, and identifying information was not shared. Audio recordings, transcripts, and coding were stored in a locked cabinet, and all data were deleted after the research process was completed. Only the findings were published, and all data were destroyed 1 year after publication.

## Results

**Figure 2.**
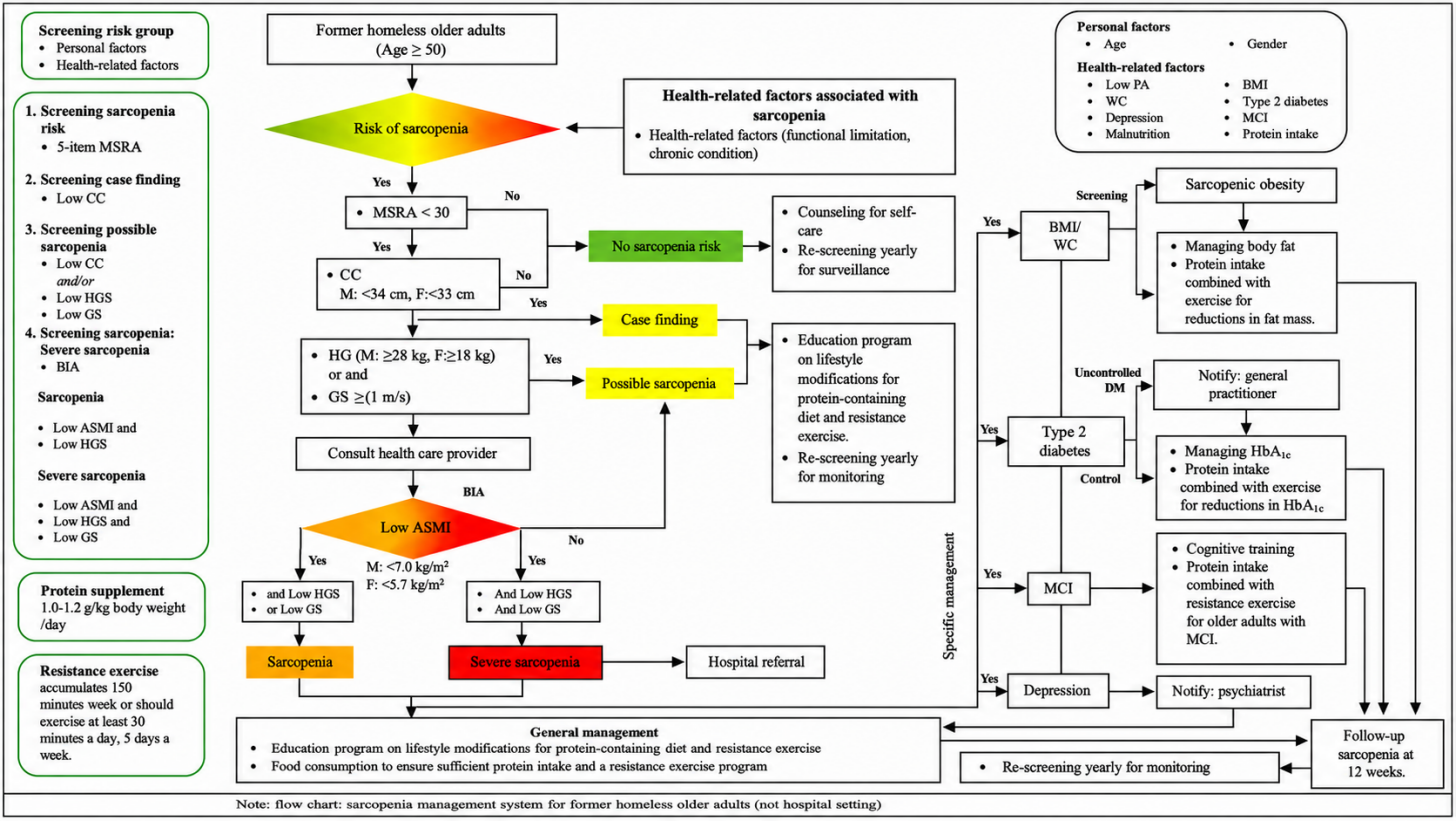
Flowchart of sarcopenia screening and management in supportive housing. ASMI: appendicular skeletal muscle mass index; BIA: bioelectrical impedance analysis; CC: calf circumference; DM: diabetes mellitus; GS: gait speed; HGS: handgrip strength; HbA_1c_: glycated hemoglobin; MCI: mild cognitive impairment; MSRA: Mini Sarcopenia Risk Assessment; PA: physical activity; VC: vital capacity; WC: waist circumference.

In phase 1, based on prior systematic reviews [23,24] and qualitative findings [21], and AWGS 2019 criteria, the sarcopenia management system (Figure 2) was developed to promote multidisciplinary collaboration among administrators, staff, and health professionals and to ensure early detection and ongoing care for older adults in supportive housing facilities. The sarcopenia management system comprises five core components: screening, classification, management, follow-up, and evaluation. Screening tools include assessments of the 5-item MSRA and health-related factors, including BMI, waist circumference, type 2 diabetes and other noncommunicable diseases, mild cognitive impairment, depression, calf circumference, handgrip strength, and gait speed, among older adults residing in supportive housing. Classification was then made into three risk levels: green (no risk), yellow (possible sarcopenia), and red (sarcopenia or severe sarcopenia), each linked to specific management strategies. A follow-up assessment for sarcopenia was scheduled 12 weeks after completion of the management program through education, structured resistance exercise, and nutritional interventions. In addition, annual rescreening was conducted to monitor long-term outcomes and progression.

In phase 2, the sarcopenia management system was implemented with the involvement of 30 stakeholders, including 1 head administrator, 2 administrators, 2 first-aid staff, 11 general staff members, 2 chefs, and 12 homeless volunteer representatives. Their ages ranged from 25 to 58 years, with most (13/18, 72.2%) between 41 and 60 years. The gender distribution was balanced (9/18, 50% male; 9/18, 50% female). The mean duration of employment was 8.22 (SD 7.73) years, with two-thirds (12/18, 66.7%) having worked for more than 5 years. Over half (10/18, 55.5%) held a bachelor’s or graduate degree, while 38.9% (7/18) had completed secondary education, and 5.6% (1/18) held a diploma. Similarly, the 12 homeless volunteer representatives had ages ranging from 36 to 73 years, with a mean age of 49.42 (SD 10.40) years. Most (8/12, 66.7%) were aged 18-49 years and were male (8/12, 66.7%). The mean duration of residence in the home for the destitute was 8.22 (SD = 7.73) years, with over half (7/12, 58.3%) having lived there for 1-3 years. One-third (4/12, 33.3%) had no formal education.

A total of 116 former homeless older adults also participated in phase 2. Their age ranged from 50 to 83 years, with a mean of 59.1 (SD 7.8) years. The majority were aged 50‐59 years (70/116, 60.3%), and more than half were male (77/116, 66.4%). Regarding the duration of former homeless older adults in the supportive housing, 55.2% (64/116) had resided there for 1‐3 years. Regarding educational attainment, nearly half of the participants completed primary school (57/116, 49.1%). Most older adults (101/116, 87.1%) reported having at least 1 chronic illness, including psychiatric disorders (71/116, 61.2%), hypertension (37/116, 31.9%), dyslipidemia (28/116, 24.1%), and type 2 diabetes (15/116, 12.9%). Regarding nutritional status, 51.7% (60/116) of participants had Short Form (MNA-SF) scores less than 12, indicating malnutrition or risk of malnutrition. Recent weight loss was reported by 15.5% (18/116) of participants, 62.9% (73/116) reported a history of smoking, while 28.4% (33/116) reported a history of falls. Regarding physical activity levels, 64.6% (75/116) were classified as sedentary, while 32.8% (38/116) engaged in moderate physical activity.

The calf circumference among participants ranged from 25.5 to 42.8 cm, with a mean of 31.51 (SD 3.16) cm. The majority of participants (93/116, 80.2%) had normal calf circumference, while 19.8% (23/116) had low calf circumference. Handgrip strength ranged from 5.8 to 42.6 kg, with a mean of 19.82 (SD 7.23) kg. Based on established cut-off values, 77.6% (90/116) of participants were classified as having low handgrip strength, whereas 22.4% (26/116) had normal handgrip strength. Gait speed ranged from 3.27 to 22.45 m/s, with a mean of 7.56 (SD 3.41) m/s. Over half of the participants (73/116, 62.9%) demonstrated normal gait speed, while 37.1% (43/116) had low gait speed.

Regarding sarcopenia risk, 19.8% (23/116) of participants were classified as having no sarcopenia risk (green zone), while 80.2% (93/116) were classified in the yellow zone through case finding. Overall, 78.4% (91/116) of participants met the AWGS 2019 criteria for possible sarcopenia. Participants in both the green and yellow zones performed resistance exercise and consumed Nile Tilapia and soy milk.

In phase 3, a preliminary evaluation was conducted to assess the sarcopenia management system’s efficiency, perceived benefits, quality, and satisfaction. Perceived benefits were high for both older adults and staff. Former homeless older adults rated system benefits with a mean of 3.8 (SD 0.4), while staff rated benefits slightly higher (mean 3.9, SD 0.3), indicating shared recognition of the system’s efficiency. The perceived quality of the sarcopenia management system among former homeless older adults achieved a mean score of 2.6 (SD 0.5). Similarly, staff rated system quality at a mean of 2.6 (SD 0.53), reflecting consistent perceptions between users and providers. Satisfaction with system processes and procedures was rated similarly (mean 2.6, SD 0.53). Overall satisfaction after system implementation was 2.8 (SD 0.4), and satisfaction with specific procedures was 2.7 (SD 0.8). These results indicate high satisfaction levels, confirming that the sarcopenia management system was both efficient and acceptable. Feedback from participants and staff suggested future refinements should focus on expanding physical activities, providing regular training, and improving follow-up monitoring for long-term sustainability.

## Discussion

### Principal Findings

This study developed and implemented a context-sensitive sarcopenia management system tailored to former homeless older adults residing in supportive housing in Thailand. Using a mixed-methods approach guided by the Donabedian framework, the system integrated structural readiness, care processes, and outcome evaluation to address sarcopenia within a constrained institutional environment. The findings demonstrate that the system is feasible, acceptable, and responsive to the specific needs of this vulnerable population.

The preliminary evaluation demonstrated that the sarcopenia management system was successfully implemented in a supportive housing setting, with 78.4% (91/116) of participants identified as having possible sarcopenia according to the AWGS 2019 criteria, and all participants completed the resistance exercise and nutritional interventions. Both formerly homeless older adults and staff reported high perceived benefits, good quality, and high satisfaction, supporting the feasibility and acceptability of the system [29].

### System Development and Contextual Relevance

The design of the sarcopenia management system reflects alignment with international clinical and public health recommendations, including those of the AWGS and European Society for Clinical Nutrition and Metabolism (ESPEN) [19,30], which emphasize early detection, resistance exercise, and adequate protein intake as core strategies for sarcopenia prevention and management. In this study, 78.4% (91/116) of participants met the criteria for possible sarcopenia [29], highlighting the substantial burden of early-stage sarcopenia in this vulnerable population and underscoring the importance of timely screening and intervention. All participants completed the resistance exercise and nutritional interventions, supporting the feasibility of implementing evidence-based sarcopenia management within supportive housing settings where resources and access to professional health services are limited. Consistent with previous evidence, integrating resistance exercise and nutritional support within the system supports muscle strength and functional maintenance among older adults [31]. Importantly, this study extends existing evidence by demonstrating how these interventions can be operationalized within supportive housing settings where resources, autonomy, and access to professional health services are limited.

The incorporation of community-based nutritional initiatives, including Nile tilapia cultivation and locally sourced soy milk for protein supplementation, represents a culturally appropriate and sustainable adaptation to the Thai supportive housing context. These strategies are supported by published evidence from this population indicating that malnutrition and low BMI are important factors associated with possible sarcopenia [29]. This approach directly addresses food security and nutritional access, which are critical social determinants of sarcopenia among former homeless older adults. Consistent with previous evidence, higher dietary quality and adequate protein intake are strongly associated with improved physical performance and functional outcomes in older adults [32,33]. The high ratings for perceived benefits, service quality, and satisfaction further suggest that these strategies were acceptable to both participants and staff and could be integrated into routine care practices. By embedding these strategies within existing community resources, the system demonstrates potential for long-term sustainability and scalability within community health settings.

In addition to alignment with AWGS and ESPEN recommendations [19,30], the developed sarcopenia management system is conceptually consistent with the World Health Organization’s Integrated Care for Older People (ICOPE) framework [34]. ICOPE emphasizes the preservation of intrinsic capacity, particularly mobility, vitality, and nutrition, as key domains for preventing functional decline in older adults [34]. The system’s focus on early sarcopenia screening, resistance exercise, nutritional support, and monitoring of functional status reflects core ICOPE principles and illustrates how intrinsic capacity-oriented care can be operationalized within supportive housing settings for former homeless older adults.

### Structural and Process Improvements Within Supportive Housing

Prior to system implementation, the supportive housing facility lacked standardized sarcopenia screening procedures and staff capacity for early identification and management. Similar challenges have been documented in other geriatric and community-based care settings, where fragmented care pathways and limited geriatric expertise compromise care quality and efficiency [35]. Mixed-methods evidence further emphasizes that the absence of structured systems and role clarity undermines preventive care delivery for older adults [35].

The implemented system addressed these gaps by introducing validated screening tools, structured protocols, and clear role delineation, enabling nonmedical staff to participate in sarcopenia prevention. In this study, 18 staff members and 12 homeless volunteers were trained to conduct sarcopenia screening and support resistance exercise and nutritional interventions, demonstrating the feasibility of task-sharing within a resource-limited setting. Reviews of sarcopenia management emphasize that multidisciplinary and multicomponent approaches are necessary to effectively address sarcopenia, as single-domain interventions are often insufficient [36,37]. By integrating screening, exercise, and nutrition into routine workflows, the present model demonstrates a pragmatic, system-level innovation that enhances efficiency without requiring additional specialist resources.

Process evaluation findings indicate that integrating screening, exercise, and nutrition into existing daily routines enhanced feasibility and adherence. All participants completed the resistance exercise and nutritional interventions, and both staff and former homeless older adults reported high perceived benefits, good service quality, and high satisfaction with the system. Stakeholder engagement and role clarification facilitated coordination across staff groups and supported consistent care delivery. These findings align with prior research demonstrating that integrated, multidisciplinary approaches improve implementation fidelity and health outcomes in long-term care environments [38,39].

### Efficiency Perceived Benefits, Quality, and Satisfaction

The preliminary evaluation demonstrated that the sarcopenia management system was feasible and well accepted among former homeless older adults residing in supportive housing. Participants and stakeholders reported high perceived benefits, good service quality, and high satisfaction, particularly regarding early detection of sarcopenia, structured follow-up, and strategies to maintain or reverse sarcopenia. These findings suggest that the system was effective in establishing a practical and organized approach to sarcopenia management within a resource-constrained institutional setting. The incorporation of nutritional interventions, including Nile tilapia cultivation and soy milk supplementation, may have contributed to these favorable perceptions by addressing modifiable nutritional factors associated with possible sarcopenia in this population [29]. The perceived benefits and high satisfaction reported by participants may be partly explained by the system’s relevance to addressing important functional vulnerabilities, including falls, mobility limitations, and loss of independence, which are common among former homeless older adults and are recognized as major consequences of sarcopenia [1,31].

Sarcopenia is a well-established risk factor for falls, functional decline, and loss of independence among older adults [1]. In the present supportive housing population, a substantial proportion of residents reported a history of falls (33/116, 28.4%), underscoring the high baseline risk and vulnerability of this group. Consistent with previous evidence, sarcopenia has been identified as an independent risk factor for falls, with fall risk increasing progressively from possible sarcopenia to sarcopenia, highlighting the importance of early screening and management for fall prevention in older adults [40].

Among populations with a history of homelessness, fall risk is further compounded by poor physical functioning, balance impairment, multimorbidity, and prolonged exposure to adverse living conditions. Prior studies among former homeless older adults living in permanent supportive housing report high fall prevalence associated with markedly impaired mobility and physical resilience [41,42]. Global falls prevention guidelines emphasize the importance of structured assessment, strength-based exercise, and mobility monitoring to reduce fall risk [43]. Systematic reviews further support the role of exercise interventions in improving muscle strength and reducing fall-related outcomes among older adults with sarcopenia [44].

By integrating routine sarcopenia screening, physical performance assessment, resistance exercise, and nutritional strategies, the developed system addresses key modifiable determinants associated with falls. Although falls were not assessed as a primary outcome in this preliminary evaluation, the system may contribute to fall prevention by promoting early detection, resistance exercise, nutritional support, and maintenance of mobility and functional capacity in this high-risk population.

### Innovation, Quality, and Responsiveness of Care

From an innovation perspective, this study differs from most existing sarcopenia interventions, which typically focus on isolated components delivered in clinical or research settings [24,31,36,37]. The proposed model represents a system-level innovation by embedding sarcopenia management into routine supportive housing workflows and enabling delivery by nonspecialist staff. This approach addresses gaps identified in prior geriatric care research, where the lack of integrated pathways and the overreliance on specialist services limit real-world applicability [35,37]. Participants also reported high satisfaction with the sarcopenia management system, reflecting positive perceptions of the screening process, exercise and nutritional components, staff support, and overall service delivery. Stakeholders similarly rated the system’s quality favorably, indicating that the program was well integrated into routine care and contributed to improved coordination, communication, and documentation.

High satisfaction and perceived benefits among both former homeless older adults and staff indicate that the sarcopenia management system was user-friendly and acceptable within the institutional context. Beyond individual-level benefits, the system contributed to improved care coordination, documentation, and communication among staff, reflecting strengthened process quality. In line with the Donabedian framework [21], improvements in structural readiness and care processes were associated with positive implementation outcomes, supporting the relevance of system-based approaches to sarcopenia management in vulnerable populations. All participants completed the resistance exercise and nutritional interventions, further supporting the system’s responsiveness and acceptability in the supportive housing setting.

### Equity, Sustainability, and Public Health Implications

The findings suggest that a structured, context-sensitive sarcopenia management system has the potential to enhance early detection, prevention, and coordinated care for institutionalized and socially marginalized older adults. By combining evidence-based interventions with locally adapted strategies, including resistance exercise, Nile tilapia cultivation, and soy milk supplementation, the system offers a practical model for supportive housing and similar community-based care settings. If implemented at scale, such systems may help maintain functional independence, reduce preventable disability, and improve equity in geriatric health services. The approach is consistent with national healthy aging priorities and the World Health Organization ICOPE framework, which emphasizes functional ability and integrated, person-centered care.

From a public health perspective, the system advances equity by extending preventive geriatric care to former homeless older adults, a population that has historically experienced limited access to coordinated health services and a high burden of functional vulnerability. In this study, 78.4% (91/116) of participants met the criteria for possible sarcopenia [29], underscoring the substantial unmet need for early identification and intervention in this underserved population. Integrated care models targeting intrinsic capacity have been shown to improve functional outcomes and support sustainable healthy aging strategies [45]. Multidisciplinary care pathways for fall prevention further underscore the importance of coordinated system-level approaches to addressing functional decline among high-risk populations [46].

By integrating care processes into existing institutional routines and using locally adapted nutritional strategies, the system supports sustainability in resource-constrained settings by minimizing reliance on external specialist resources and strengthening staff capacity within supportive housing facilities.

### Strengths and Limitations

This study is among the first to develop and pilot a sarcopenia management system specifically for former homeless older adults residing in supportive housing. The use of a Donabedian-guided mixed-methods design strengthened methodological rigor by linking structural conditions, care processes, and outcomes. Extensive stakeholder involvement enhanced feasibility, acceptability, and real-world applicability. However, the study was conducted in a single facility, and the sample size was limited to a preliminary feasibility and acceptability assessment. Long-term outcomes and cost-effectiveness were not evaluated. Future studies should use multisite designs with longitudinal follow-up to assess sustainability, effectiveness, and economic implications. In addition, potential confounding factors, such as age, nutritional status, physical activity, and comorbidities, were not statistically controlled due to the preliminary design and descriptive analytical approach. Future studies should include larger samples and apply multivariable analyses to examine the effects of the sarcopenia management system while controlling for these factors.

### Conclusions

This study demonstrated the feasibility and acceptability of a context-sensitive sarcopenia management system for former homeless older adults residing in supportive housing in Thailand. By integrating evidence-based screening, resistance exercise, and nutritional support within an institutional care framework, the system addressed both clinical and social dimensions of sarcopenia management in a highly vulnerable population. The findings suggest that this structured, stakeholder-supported approach may strengthen early detection and coordinated care and support the maintenance of functional ability and independence for older adults in supportive housing settings. Further research is warranted to evaluate long-term effectiveness, sustainability, scalability, and cost-effectiveness prior to broader implementation.

## Abbreviations

AWGS: Asian Working Group for Sarcopenia
BIA: bioelectrical impedance analysis
CMT: Chula Mental Test
CVI: content validity index
ESPEN: European Society for Clinical Nutrition and Metabolism
ICOPE: Integrated Care for Older People
MSRA: Mini Sarcopenia Risk Assessment
